# Novel prognostic model for stratifying survival in stage I lung adenocarcinoma patients

**DOI:** 10.1007/s00432-019-03110-y

**Published:** 2019-12-28

**Authors:** Di-Han Liu, Zheng-Hao Ye, Si Chen, Xue-Song Sun, Jing-Yu Hou, Ze-Rui Zhao, Hao Long

**Affiliations:** 1 State Key Laboratory of Oncology in Southern China, Collaborative Innovation Center for Cancer Medicine, and Department of Thoracic Surgery, Sun Yat-Sen University Cancer Center, 651 Dongfeng Road East, Guangzhou, 510060 People’s Republic of China; 2 Lung Cancer Research Center, Sun Yat-Sen University, Guangzhou, People’s Republic of China; 3 The Third Affiliated Hospital of Guangzhou Medical University, Guangzhou, People’s Republic of China; 4 State Key Laboratory of Oncology in Southern China, Collaborative Innovation Center for Cancer Medicine, and Department of Nasopharyngeal Carcinoma, Sun Yat-Sen University Cancer Center, 651 Dongfeng Road East, Guangzhou, 510060 People’s Republic of China

**Keywords:** Adenocarcinoma, Prognosis, Tumor necrosis, Architectural grade

## Abstract

**Purpose:**

We combined conventional clinical and pathological characteristics and pathological architectural grading scores to develop a prognostic model to identify a specific group of patients with stage I lung adenocarcinomas with poor survival following surgery.

**Methods:**

This retrospective study included 198 patients with stage I lung adenocarcinomas recruited from 2004 to 2013. Multivariate analyses were used to confirm independent risk factors, which were checked for internal validity using the bootstrapping method. The prognostic scores, derived from β-coefficients using the Cox regression model, classified patients into high- and low-risk groups. The predictive performance and discriminative ability of the model were assessed by the area under the receiver operating characteristic curve (AUC), concordance index (C-index) and Kaplan–Meier survival analyses.

**Results:**

Three risk factors were identified: T2 (rounding of β-coefficients = 81), necrosis (rounding of β-coefficients = 67), and pathological architectural score of 5–6 (rounding of β-coefficients = 58). The final prognostic score was the sum of points. The derived prognostic scores stratified patients into low- (score ≤ 103) and high- (score > 103) risk groups, with significant differences in 5-year overall survival (high vs. low risk: 49.3% vs. 88.0%, respectively; hazard ratio: 4.55; *p* < 0.001). The AUC for the proposed model was 0.717. The C-index of the model was 0.693.

**Conclusion:**

An integrated prognostic model was developed to discriminate resected stage I adenocarcinoma patients into low- and high-risk groups, which will help clinicians select individual treatment strategies.

**Electronic supplementary material:**

The online version of this article (10.1007/s00432-019-03110-y) contains supplementary material, which is available to authorized users.

## Introduction

Lung cancer is the leading cause of cancer death in males and the second leading cause of cancer death in females worldwide (Torre et al. 2016). The 5-year survivals of patients with pathological stage IA after surgery are 92%, 86%, and 81% for stages IA1, IA2, and IA3, respectively (Nowak et al. 2016). Among early-stage patients, 23–29.1% develop recurrence despite curative resection (Kelsey et al. 2013; Taylor et al. 2012). According to the American Society of Clinical Oncology adjuvant therapy guideline for resected non-small-cell lung cancers (NSCLCs), adjuvant chemotherapy is recommended for patients with stage IIA, IIB, or IIIA disease who have undergone complete surgical resection (Kris et al. 2017). However, the indications for postoperative chemotherapy for stage I patients are still controversial (Bradbury et al. 2017). The decision of which stage IB patients to treat with adjuvant chemotherapy is not as clear as in other stages. Additional prognostic markers beyond stage are needed to determine who may be in need of adjuvant chemotherapy or more aggressive treatment approach.

Previous studies have considered various assessment methods, including grading systems based on certain pathological, architectural, or pathological characteristics, and genomic profiling, for investigating stage I NSCLC patients with a high chance of early relapse (Zhao et al. 2015; Kadota et al. 2012; Ooki et al. 2017; Kratz and Jablons 2009). In the present study, we constructed a novel but concise prognostic model based on conventional clinical and pathological characteristics to stratify patients who underwent complete anatomical resection into different risk groups for developing early recurrence. Using the model, we were able to identify a subset of stage I patients with a higher risk of recurrence and poor survival who may be in need of more aggressive adjuvant treatments or closer follow-up strategies.

## Materials and methods

### Patients

This study enrolled 198 patients who underwent anatomical resection with systematic lymph node dissection using thoracotomy or video-assisted thoracic surgery, and who were pathologically diagnosed with stage IA or IB invasive lung adenocarcinomas according to the 8th edition staging system (Detterbeck et al. 2017) for lung cancer from 2004 to 2013 at Sun Yat-Sen University Cancer Center, Guangzhou, China. The patients’ clinical information, pathological findings, and prognoses obtained from the hospital database were evaluated retrospectively. The patients were subsequently followed up every 3 months during the first 2 years, every 6 months during the next 3 years, and then annually. Routine chest and upper abdominal computed tomography, with cranial magnetic resonance imaging or positron emission tomography, if applicable, was performed to evaluate the postoperative recurrence during the follow-up. The study protocol was approved by the institutional review board. The IRB approval number was 2013-FXY-048-Department of thoracic.

### Morphological evaluation and grading system

Invasive adenocarcinomas were classified as lepidic (LEP), acinar (ACN), papillary (PAP), micropapillary (MIP), and solid (SOL) according to the 2015 World Health Organization classification for lung cancer (Travis et al. 2015). The architectural grading system divided growth patterns into three major categories: grade 1 for LEP, grade 2 for ACN and PAP, and grade 3 for MIP and SOL (Zhao et al. 2015). Patients with a pure growth pattern were given an identical grade for both the first and second grades. The final architectural score was the sum of the grades of the two most predominant patterns. The predominant pattern was defined as that present in the highest percentage within the tissue, with the lowest limit set at 30%. Vascular and/or lymphatic invasion and tumor necrosis were observed. The presence of neuron invasion was defined as tumors involving the epineurium in the peri-tumoral tissue. Two pathologists worked together using a multi-headed microscope, and discussed the analyses until agreement was reached.

### Statistical analyses

Overall survival (OS) was defined as the time of surgery to death. Disease-free survival (DFS) was defined as the interval between resection and the first recurrence. Patients without an event were censored at the end of the follow-up. The survival curves were estimated and compared using the Kaplan–Meier method and log-rank test. Receiver operating characteristic (ROC) curve analyses were adopted for dichotomization according to the DFS. The ROC analyses were used to assess the predictive accuracy of the prognostic model using the area under the curve (AUC) determination. Comparison of paired AUROCs and 95% confidence intervals (CIs) was performed using the nonparametric Delong test. The predicting performance of the model was also evaluated by calculating the concordance index (C-index) which ranges from 0.5 to 1.0, with 0.5 indicating a random chance and 1.0 indicating a perfect ability to correctly discriminate the outcome with the model. Variables with a value of *p* < 0.05 in univariate analyses were subsequently entered into a Cox regression model for multivariate analyses using the backward conditioned method with estimation of the corresponding hazard ratio (HR), 95% confidence interval (CI), and probability *p* values. All reported *p* values were two tailed, with *p* < 0.05 considered statistically significant. Statistical analyses were performed using SPSS statistical software for Windows, version 24.0 (IBM, Armonk, NY, USA) and R (https://www.R-project.org) version 3.5.2.

### Model development

Patient demographics (age and sex) and clinical and pathological parameters (smoking history, type of surgery, adjuvant chemotherapy, T stage, lymphovascular invasion, necrosis, neuron invasion, and architectural score) were analyzed for possible correlations with the DFS (as more incidents occurred in stage I patients who had received anatomical resection with systematic lymph node dissection, it may better represent the prognoses) using the Cox proportional HR. Covariates with *p* < 0.05 were included in the multivariate model to build the scoring system. The bootstrapping method, resampled to *n* = 1000, was used to check the internal validity and stability of the Cox regression equation (Sauerbrei and Schumacher 1992). The β-coefficient of the respective log(HR) obtained from the multivariate model was used to derive weighting factors of the prognostic score that were equal to 100* β-coefficient with rounding. The final prognostic score was the sum of individual scores. The ROC analyses were adopted for the dichotomization of the prognostic score according to outcomes to determine the best splitter threshold.

## Results

### Patient demographics and survival

The baseline characteristics of the cohort are summarized in Table 1. In total, only 35 patients (17.7%) had a pure tumor growth pattern (6 for MIP/SOL and 29 for ACN/ PAP) and 163 patients (83.2%) presented with mixed growth patterns. There were 89 (44.9%), 31 (15.7%) and 72 (36.4%) patients who scored 3, 4 and 5 in pathological architectural score, respectively. None of the 198 patients received neoadjuvant chemotherapy. The median follow-up interval was 44.67 months (range 6.23–101.53 months), and 73 patients (36.1%) had documented recurrence and 33 patients (16.7%) died during follow-up. The 5-year OS and DFS across the entire cohort were 78.7% and 57.8%, respectively.

**Table 1:** The baseline characteristics of patients

Characteristics	Number of patients (%)
Age, years (range)	
Median (range)	60.4 (29–81)
Gender	
Male	95 (48.0%)
Female	103 (52.0%)
Smoking history	
No	122 (61.6%)
Yes	76 (38.4%)
T stage^a^	
T1	126 (63.6%)
T2	72 (39.4%)
Architectural score	
3–4	120 (60.6%)
5–6	78 (39.4%)
Lymphovascular invasion	
No	111 (56.1%)
Yes	87 (43.9%)
Neuron invasion	
No	179 (90.4%)
Yes	19 (9.6%)
Necrosis	
No	154 (77.8%)
Yes	44 (22.2%)
Type of surgery	
Open	143 (72.2%)
VATS	55 (27.8%)
Adjuvant chemotherapy	
No	148 (74.7%)
Yes	50 (25.3%)

*VATS* video-assisted thoracic surgery

^a^According to the 8th edition of UICC/AJCC staging system

### Prognostic scoring system

To develop the model, we first tested the covariates listed in Table 1 for their association with DFS in the cohort. Significant predictors of DFS in univariate analyses are shown in Table 2. Smoking history, sex, T stage, lymphovascular invasion, necrosis, and pathological architectural scores were included in the multivariate analyses. Then the bootstrapping method (resampled to *n* = 1000) was performed, which resulted in highly consistent and stabile risk factors (Table 2). In summary, T stage (HR: 2.25; 95% CI 1.41–3.60; *p* = 0.001), necrosis (HR: 1.95; 95% CI 1.15–3.30; *p* = 0.013), and architectural score (HR: 1.79; 95% CI 1.09–2.94; *p* = 0.021) were the key prognostic predictors. Then a scoring model based on weighting (derived by the β-coefficient of the respective log[HR]) of the three significant covariates was constructed. The score for each covariate is listed in Table 3.

**Table 2:** Univariate and multivariate analyses of disease-free survival after internal validation by bootstrapping method

Characteristics	Univariate analyses		Multivariate analyses	
	Hazard ratio (95% CI)	*P*	Hazard ratio (95% CI)	*P*
Age, years (range)				
< 70 (29–70)	Reference			
≥ 70 (70–81)	0.96 (0.54–1.69)	0.885		
Gender				
Male	Reference		Reference	
Female	0.51 (0.32–0.82)	**0.005**	0.71 (0.37–1.39)	0.317
Smoking history				
No	Reference		Reference	
Yes	1.86 (1.18–2.95)	**0.008**	1.05 (0.54–2.03)	0.895
T stage^a^				
T1	Reference		Reference	
T2	2.20 (1.39–3.49)	**0.001**	2.25 (1.41–3.60)	**0.001**
Architectural score				
3–4	Reference		Reference	
5–6	2.26 (1.42–3.59)	**0.001**	1.79 (1.09–2.94)	**0.021**
Lymphovascular invasion				
No	Reference		Reference	
Yes	2.54 (1.59–4.08)	**< 0.001**	1.66 (0.97–2.85)	0.064
Neuron invasion				
No	Reference			
Yes	1.69 (0.87–3.30)	0.124		
Necrosis				
No	Reference		Reference	
Yes	2.84 (1.76–4.59)	**< 0.001**	1.95 (1.15–3.30)	**0.013**
Type of surgery				
Open	Reference			
VATS	0.90 (0.53–1.53)	0.697		
Adjuvant chemotherapy				
No	Reference			
Yes	1.12 (0.67–1.86)	0.674		

All statistically significant variables are highlighted in bold

Hazard ratios estimated by Cox proportional hazards regression. All statistical tests were two sided

*VATS* video-assisted thoracic surgery

^a^According to the 8th edition of UICC/AJCC staging system

**Table 3:** Constructed prognostic score to predict DFS in patients with adenocarcinoma

Variables	Hazard ratio	*β* [HR = exp(β)]	Score^b^
T stage^a^			
T1	1	0	0
T2	2.254	0.813	81
Architectural score			
3–4	1	0	0
5–6	1.792	0.583	58
Necrosis			
No	1	0	0
Yes	1.951	0.668	67

Hazard ratios estimated by Cox proportional hazards regression

^a^According to the 8th edition of UICC/AJCC staging system

^b^Score = 100*beta with rounding

**Fig. 1:**
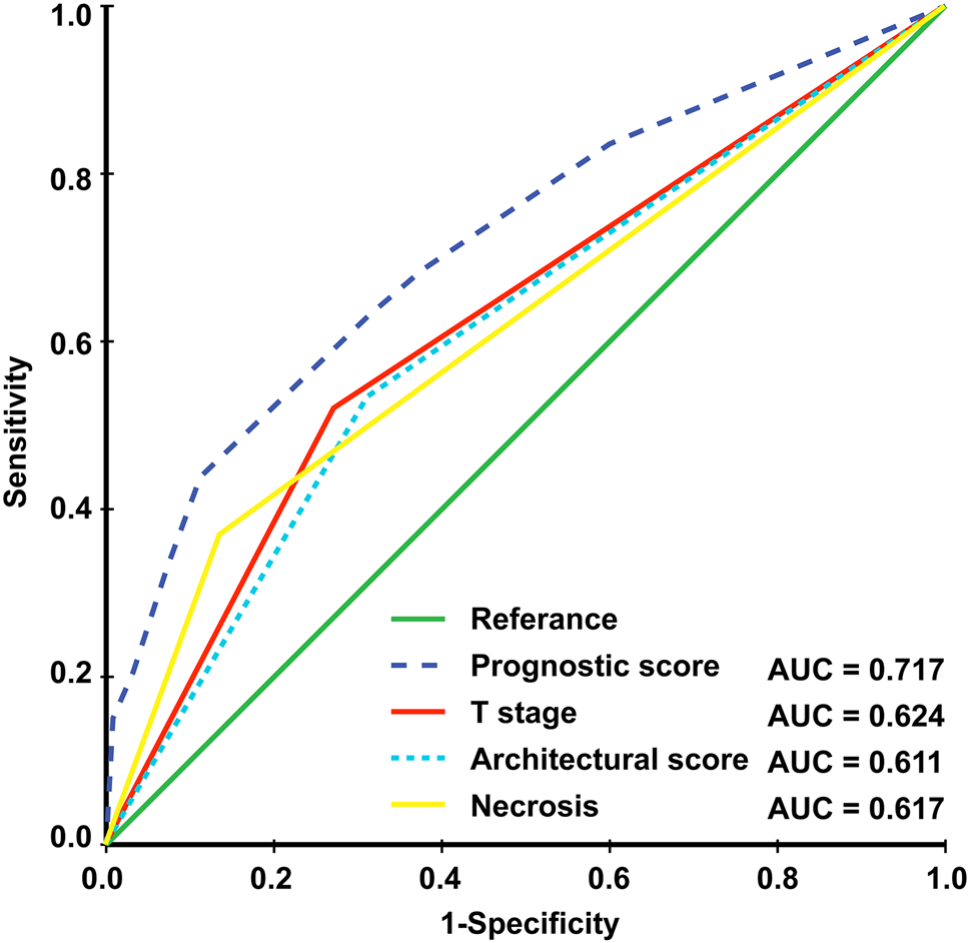
Receiver operating characteristic curve and accuracy of the prognostic score system, architectural score, T stage and necrosis

**Fig. 2:**
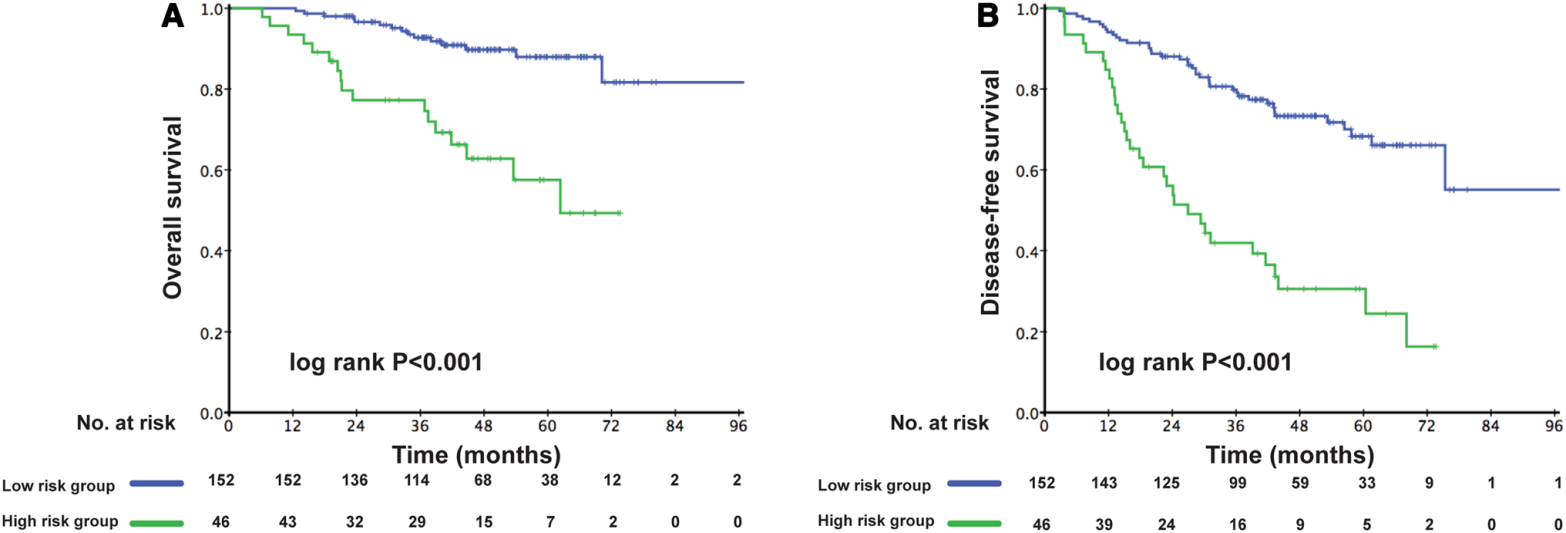
Kaplan–Meier curves of low-risk and high-risk subgroups of stage I adenocarcinoma patients for overall survival (**a**) and disease-free survival (**b**)

**Fig. 3:**
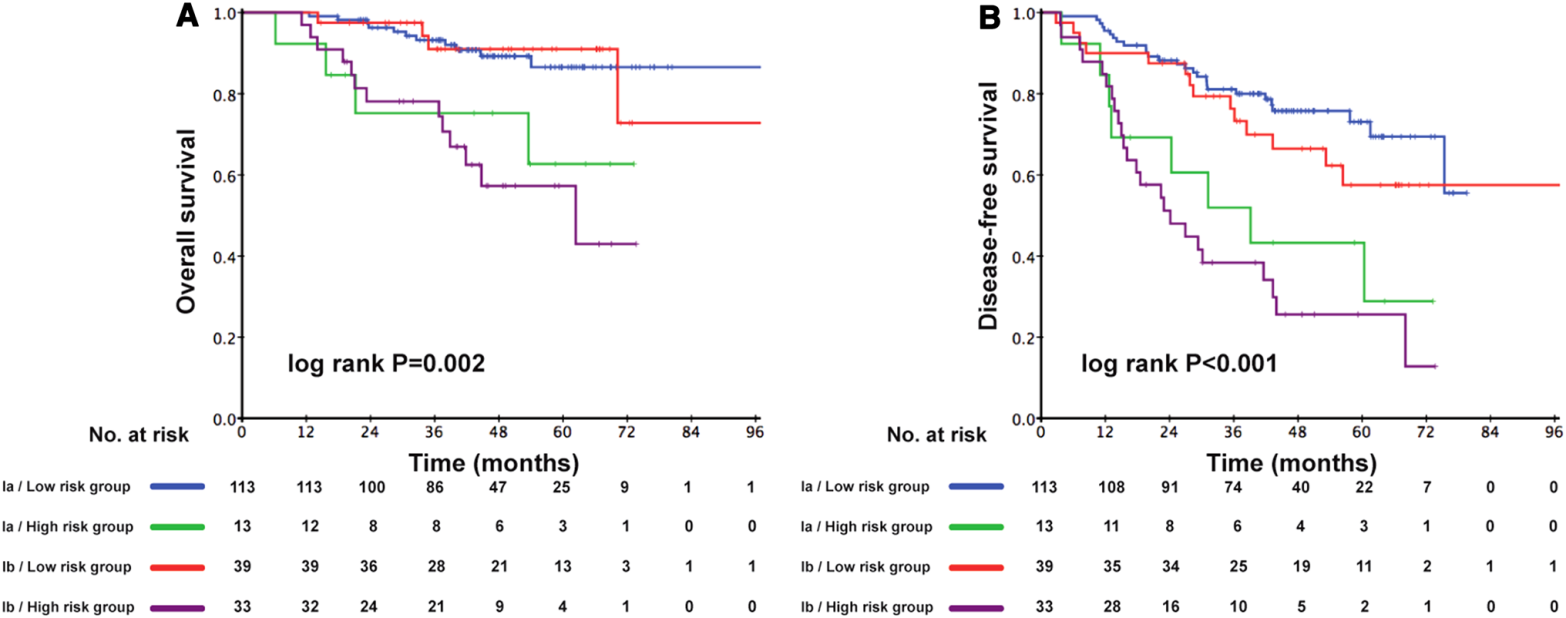
Kaplan–Meier curves of low-risk and high-risk subgroups of stage Ia and Ib adenocarcinoma patients for overall survival (**a**) and disease-free survival (**b**)

The prognostic score (range 0–206) was calculated for each patient. Dichotomization into the low- and high-risk subgroups was based on a cutoff value determined by the ROC analyses predicting the outcomes of 103 points. The AUC for the prognostic score model was 0.717, which was greater than the T stage (AUC: 0.624), architectural score (AUC: 0.611), and necrosis (AUC: 0.617) as shown in Fig. 1. The 95% confidence interval of AUC and the *p* value are listed in Supplement Table 1. The C-index for the established model to predict DFS was 0.693. Kaplan–Meier curves revealed a significant survival difference between the low- and high-risk subgroups in both OS and DFS across the entire cohort (Fig. 2). As shown in Fig. 3, the risk model stratified the Kaplan–Meier curves for OS and DFS into the low- and high-risk subgroups of stages Ia and Ib. Importantly, the pathological stage (Ia and Ib) did not differentiate survival curves within a single-risk group.

## Discussion

Patients with early-stage NSCLC after complete surgical resection are at substantial risk for recurrence. The role of adjuvant chemotherapy for stage I patients is still controversial because previous randomized trials have not reported consistent results (Kris et al. 2017; Bradbury et al. 2017). In daily practice, the tumor–node–metastasis (TNM) system is used to differentiate the prognoses of patients with NSCLC. However, patients with the same TNM stage may have completely different outcomes (Chansky et al. 2009). Because of the heterogeneous nature of NSCLC, it would be imprecise to predict survival using the TNM staging system alone. For this reason, various studies have identified early-stage NSCLCs with poor survival that could potentially benefit from adjuvant treatment.

In our study, the presence of necrosis had significant prognostic implications, in line with previous studies (Makinen et al. 2017; Qian et al. 2018; Yi et al. 2018; Li et al. 2017; Yoshizawa et al. 2011). One of the possible reasons for this may be that the process of tumor necrosis may release proinflammatory intracellular contents into the tumor microenvironment, inducing an inflammatory response involving a diverse set of immune cells such as neutrophils. Tumor-associated neutrophils are associated with poor prognoses in a variety of cancer types, and a previous study reported consistently increased levels of necrosis and infiltration of neutrophils (Li et al. 2017). Another explanation is that most SOL and MIP adenocarcinomas are correlated with the presence of tumor necrosis, with SOL adenocarcinomas showing the highest indices (Makinen et al. 2017). Similar results were found in our study; in 44 adenocarcinomas with necrosis, 25 had SOL and MIP patterns (*p* = 0.009).

Yoshizawa et al. (2011) proposed three architectural grades: low (LEP), intermediate (ACN and PAP), and high grade (SOL and MIP) based on the predominant growth patterns of invasive carcinomas. In our study, there are no patients with pure lepidic growth pattern. However, most lung adenocarcinomas have mixed growth patterns. A combination of high-grade parts could result in more aggressive biological behavior. Sica et al. (2010) proposed a grading system to stratify prognostic differences in early-stage adenocarcinomas by integrating the two most representative grades of a tumor, which provided a more comprehensive description of tumor aggressiveness. In a previous study (Zhao et al. 2015), we validated the prognostic effects of the grading system for stage I adenocarcinomas in an Asian population. In addition, patients with high-grade components were at higher risk for local–regional recurrence following sublobar resection, so adjuvant therapy may be needed for these patients, even in the early stages (Zhao et al. 2018). Kadota et al. (2012) proposed a grading system that combined the predominant growth pattern and the mitotic count in ten high-power fields (HPFs). Adenocarcinomas with an intermediate architectural grade (ACN or PAP) with a low (< 3/10 HPFs) mitotic count were classified as low grade. Intermediate architectural grade with intermediate–high mitotic counts was considered intermediate grade and high architectural grade with any mitotic count was classified as high grade. The combination of the architectural grade and the mitotic count also stratified stage I lung adenocarcinomas into different risk groups of recurrence. However, Barletta et al. (2010) did not recognize mitotic count as a significant prognostic factor.

Previous studies of prognostic signatures of genetic expressions (Shedden et al. 2008; Chen et al. 2007; Kratz et al. 2012; Wistuba et al. 2013) have identified two molecular prognostic markers for lung adenocarcinoma (Kratz et al. 2012; Wistuba et al. 2013), but their accuracy in predicting survival is limited. Li et al. (2017) proposed an individualized prognostic signature for non-squamous NSCLC based on immune-related gene pairs along with clinical factors, but its clinical utility also needs to be further tested and validated. Martinez-Terroba et al. (2018) proposed a prognostic signature based on three proteins (BRCA1, QKI, and SLC2A1) to stratify early-stage lung adenocarcinoma patients. Taken together, these studies have illustrated the importance of genetic aspects in predicting prognoses of lung adenocarcinomas. However, considering the additional cost of various genes selected from different studies, which may not be generalized in different populations, the actual clinical utilization of these genetic profiling methods still remains limited in daily clinical practice.

In this study, we identified a novel and practical prognostic model based on morphological features and the TNM stage of patients who underwent anatomical resection of stage I adenocarcinomas. The predictive ability of this model is higher than that of TNM staging and pathological architectural score, with a greater AUC. Stratifying pathological stage I patients into high- and low-risk subgroups for predicting early relapses might have an important impact on individualized treatment strategies.

The limitations of our study included its retrospective nature and the small size of the cohort, which also prevented us from developing an external validation cohort. The model also failed to incorporate other clinical and pathological prognostic factors (e.g., tumor markers, PET–CT value, and central or peripheral tumor) and some important recognized prognostic molecular factors (e.g., KRAS mutations, EGFR mutations, and ALK rearrangements). Incorporation of other prognostic factors may improve this model. Further studies are also needed to validate this prognostic model to assess its real efficacy and to better analyze its utility in clinical practice and trials.

In conclusion, we developed a prognostic model based on morphological features and TNM stage for stage I lung adenocarcinoma patients. We were able to identify low- and high-risk subgroups of stage I patients after surgery, which may help clinicians select individual treatments and management strategies.

## Electronic supplementary material

Below is the link to the electronic supplementary material.

Figure S1 Kaplan–Meier curves of stage Ia and Ib adenocarcinoma patients for Overall survival (A) and Disease-free survival (B)

Supplementary file2 (DOCX 14 kb)
