# Supplementary figures and images for: Novel prognostic model for stratifying survival in stage I lung adenocarcinoma patients
Source: J Cancer Res Clin Oncol. 2019 Dec 28;146(3):801–7. doi: 10.1007/s00432-019-03110-y (PMC7040084; doi:10.1007/s00432-019-03110-y)

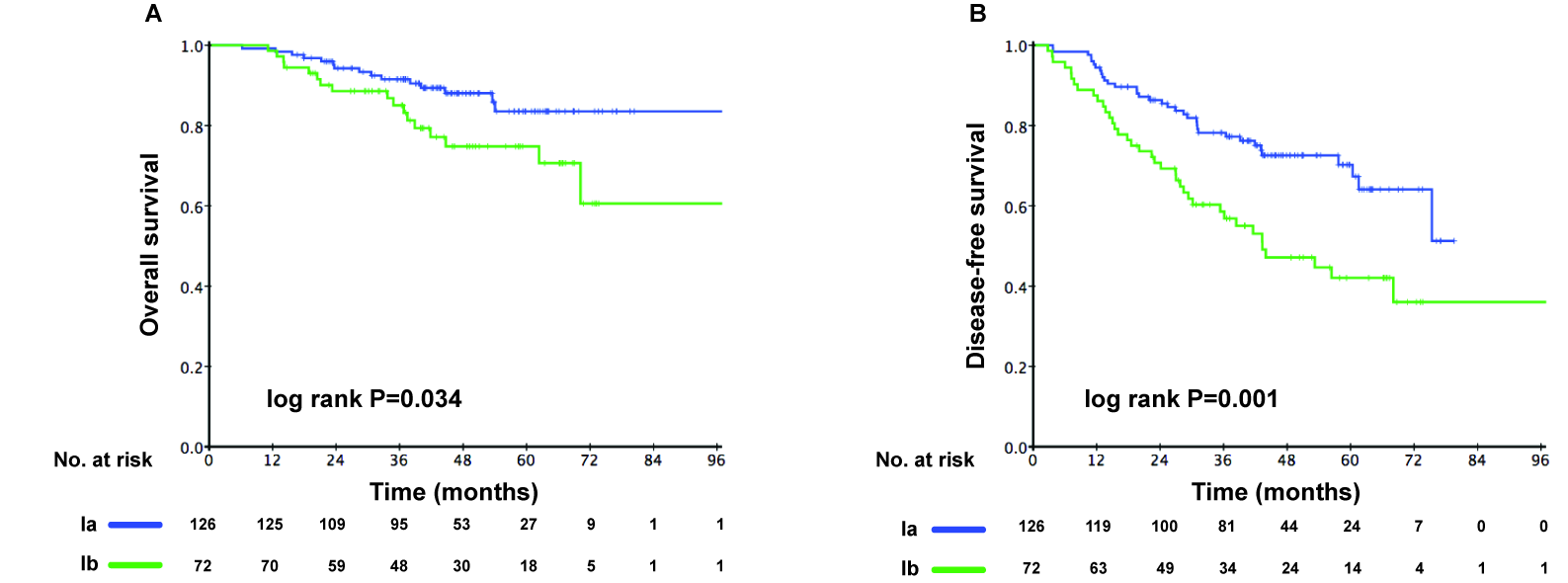

Supplement: Supplementary file 1 — Figure S1 Kaplan–Meier curves of stage Ia and Ib adenocarcinoma patients for Overall survival (A) and Disease-free survival (B) [file 432_2019_3110_MOESM1_ESM.tif]
