# Supplementary material for: Novel prognostic model for stratifying survival in stage I lung adenocarcinoma patients
Source: J Cancer Res Clin Oncol. 2019 Dec 28;146(3):801–7. doi: 10.1007/s00432-019-03110-y (PMC7040084; doi:10.1007/s00432-019-03110-y)
Supplement: Supplementary file 2 — Supplementary file2 (DOCX 14 kb) [file 432_2019_3110_MOESM2_ESM.docx]

Table S1: The AUC of T stage, architectural score, necrosis and prognostic score for prediction

| **Variable** | AUC (95% CI) | P value |
| --- | --- | --- |
| T stage | 0.624(0.583-0.665) |  |
| architectural score | 0.611(0.570-0.653) |  |
| Necrosis | 0.617(0.575-0.660) |  |
| Prognostic score | 0.717(0.679-0.755) |  |
| Prognostic score vs. T stage   \| Prognostic score \|  \| \| --- \| --- \| | | 0.042 |
| Prognostic score vs. Architectural score | | 0.022 |
| Prognostic score vs. Necrosis | | 0.033 |

*****According to the 8th edition of UICC/AJCC staging system

P values are calculated based on Z test
